# Integration of enzyme constraints in a genome-scale metabolic model of *Aspergillus niger* improves phenotype predictions

**DOI:** 10.1186/s12934-021-01614-2

**Published:** 2021-06-30

**Authors:** Jingru Zhou, Yingping Zhuang, Jianye Xia

**Affiliations:** 1 State Key Laboratory of Bioreactor Engineering, East China University of Science and Technology, Shanghai, 200237 China; 2 Tianjin Institute of Industrial Biotechnology, Chinese Academy of Science, Tianjin, 300308 China

**Keywords:** Genome-scale metabolic model, Proteome constraint GSMM, *Aspergillus niger*, Differential expression of enzymes

## Abstract

**Background:**

Genome-scale metabolic model (GSMM) is a powerful tool for the study of cellular metabolic characteristics. With the development of multi-omics measurement techniques in recent years, new methods that integrating multi-omics data into the GSMM show promising effects on the predicted results. It does not only improve the accuracy of phenotype prediction but also enhances the reliability of the model for simulating complex biochemical phenomena, which can promote theoretical breakthroughs for specific gene target identification or better understanding the cell metabolism on the system level.

**Results:**

Based on the basic GSMM model iHL1210 of *Aspergillus niger*, we integrated large-scale enzyme kinetics and proteomics data to establish a GSMM based on enzyme constraints, termed a GEM with Enzymatic Constraints using Kinetic and Omics data (GECKO). The results show that enzyme constraints effectively improve the model’s phenotype prediction ability, and extended the model’s potential to guide target gene identification through predicting metabolic phenotype changes of *A. niger* by simulating gene knockout. In addition, enzyme constraints significantly reduced the solution space of the model, i.e., flux variability over 40.10% metabolic reactions were significantly reduced. The new model showed also versatility in other aspects, like estimating large-scale $k_{{cat}}$ values, predicting the differential expression of enzymes under different growth conditions.

**Conclusions:**

This study shows that incorporating enzymes’ abundance information into GSMM is very effective for improving model performance with *A. niger*. Enzyme-constrained model can be used as a powerful tool for predicting the metabolic phenotype of *A. niger* by incorporating proteome data. In the foreseeable future, with the fast development of measurement techniques, and more precise and rich proteomics quantitative data being obtained for *A. niger*, the enzyme-constrained GSMM model will show greater application space on the system level.

**Supplementary Information:**

The online version contains supplementary material available at 10.1186/s12934-021-01614-2.

## Background

*Aspergillus niger* (*A. niger*) is widely used in industrial fermentation for producing citric acid and glucoamylase as it is approved as Generally Regarded As Safe (GRAS) [1–4]. In the past two decades, Genome-scale metabolic model (GSMM) of *A. niger* has been proposed and continuously updated [5–8], and has shown its versatility for simulating physiological properties of *A. niger*, such as exploring the relationship between environmental pH and acid production [9], predicting product yield [1, 10], etc. The GSMM has broadened the study of microbial metabolism characteristics from a small local pathway to the entire metabolic network in a systematical way. However, due to a lack of full understanding of the expression regulatory network, GSMM is still far from representing all the real cell physiological properties. The multi-omics data actually contains more information about the physiological characteristics of microbial cells. As researchers explore the physiological characteristics of *A. niger*, great progress has been made for proteomics study on *A. niger* [11–13]. Enzymes are the key players of metabolic reactions, as they play the catalyzing function and their expression levels determine the maximum flux of individual reaction. Therefore, it is natural to take enzyme abundance data as constraints for GSMM, which may improve the prediction accuracy of the model. The idea implemented on *Saccharomyces cerevisiae* has been proven effective [14].

Initially, enzyme constraints to GSMM were implemented mainly based on transcriptome data, i.e., using mRNA level instead of protein level as upper bound for corresponding reaction flux [15]. Or implementing constraint to the GSMM by restricting the total concentration of enzymes based on limitation of cytoplasmic space, such as FBAwMC [16]. However, simple ON/OFF constraints or total enzyme concentration constraints are not sufficient to capture the detailed relationship between enzyme abundance and the flux of enzyme-catalyzed reactions, so kinetics and protein allocation must be taken into consideration, like integrative omics-metabolic analysis (IOMA) [17]. IOMA is a method that combined GSMM with Michaelis–Menten form-like enzyme kinetics to estimate the reaction flux of the central pathway. It provided a reference formula to characterize the relationship between reaction flux and enzyme concentration from the perspective of kinetics, and it was successfully used to predict the metabolic flux for engineered human red blood cells with gene knockout. Resource Balance Analysis (RBA) [18] took protein allocation into account for the construction of GSMM. This method used multiple experimental sets data to estimate the apparent catalytic rate of enzymes, and it applied the estimated value as a hard constraint ($\left| {v_{i} } \right| = k_{{E_{i} }}  \times E_{i}$) to predict protein allocation. In the simulation of *Bacillus subtilis*, the allocation of bacterial protein resources was accurately and effectively predicted [18]. Recently, the allocation rules of the proteome have been further integrated into GSMM by dividing the whole cell proteome into four functional blocks [19]. This model revealed the relationship between proteome and metabolism: the maximum biomass specific growth rate may be determined by a large number of growth-related proteomes and their synthesis cost, and the affinity between the enzyme and the substrate (Michaelis–Menten constant in reaction kinetics) is related to the growth kinetic constant (Monod constant in growth kinetics), which depends on cellular metabolism strategy. In addition, more flexible soft constraints (inequalities) have also made some progress like GECKO [14]. GECKO used enzyme utilization and $k_{{cat}}$ value to expand the stoichiometric matrix S so that the model can integrate quantitative proteomics data more directly and easily. GECKO has shown good flexibility and more accurate prediction ability, which has been confirmed in *yeast*, *Bacillus subtilis,* and *Escherichia coli* [14, 20, 21].

In this study, the GECKO [14] was extended to apply to the GSMM of *A. niger*—the enzyme kinetic parameters ($k_{{cat}}$) and protein abundance were correlated with the metabolic reaction to achieve the global integration of large-scale proteomics data with GSMM, which aimed to improve the metabolic prediction accuracy for *A. niger* and to seek for the potential genetic target to improve properties of the strain, e.g., high yield, high productivity, etc. We conducted a comprehensive evaluation of the enzyme-constrained GSMM model and confirmed that it has better prediction accuracy than traditional GSMM. We further prove that the model can simulate phenotype variation caused by gene knockout from the perspective of enzymes. Furthermore, the model can predict the requirement of differential expression of proteins under different substrate conditions, which allows interpretation of the metabolic phenotype shifts on the proteomics level.

## Methods

### Integration of enzyme data with GSMM

The *A. niger* GSMM model iJB1325 [8], including 2320 reactions, 1818 metabolites, and 1325 genes, was used as the basic model. Based on iJB1325, the enzyme-constrained GSMM was constructed following the GECKO [14] method through integrating the kinetic parameters and abundance information of enzymes. The newly established model hereinafter was referred to as eciJB1325. It integrates 1255 enzymes’ kinetics and abundance into the GSMM iJB1325 of *A. niger*. Both kinetic parameter ($k_{{cat}}$) and the enzyme abundance data are used to restrict the reaction flux. As in Eq. 1,

1 $$ v_{j}  \le k_{{cat}}^{j}  \times \left[ {E_{j} } \right] $$

In Eq. 1, $v_{j}$ represents the flux of the jth metabolic reaction, and $\left[ {E_{j} } \right]$ is the concentration of the corresponding enzyme that catalyzes the reaction. The underlying rationality of this equation is that the actual reaction rate must be less than the maximum reaction rate determined by the product of $k_{{cat}}$ and enzyme concentration.

Based on the above principles, without changing the linear structure of iJB1325, we extended the stoichiometric matrix of the model according to the following steps:

i. Convert the iJB1325 to an irreversible reaction model, and then the total number of reactions was increased from 2320 to 3030.
ii. Treat enzymes as metabolites in reactions, and its stoichiometry is the reciprocal of $k_{{cat}}$ value, for example, A + 1/$k_{{cat}}$ enzyme → B + C. And add an exchange reaction for all enzymes respectively.
iii. Introduce 574 pseudo-metabolites for distinguishing isozymes. For instance, A reaction can be catalyzed by two isozymes, A → B, can be rewritten as three reactions: A → pseudo-metabolite, pseudo-metabolite + 1/$k_{{cat,1}}$ isozyme1 → B, pseudo-metabolite + 1/$k_{{cat,2}}$ isozyme2 → B.
iv. Set the upper limit of enzyme-exchange reaction as the abundance of enzyme.

Constraints with reversible enzymes (enzymes that catalyze reversible reaction) and multifunctional enzymes (enzymes that have ability to catalyze more reactions with the same enzyme) are the same as general enzymes, it should be noted that for the reversible enzymes, the reversible reactions have been splited into two irreversible reactions, with the same enzyme assigned with two different $k_{{cat}}$ values to represent the different catalyze activity for the two reactions respectively; For multifunctional enzymes, specific $k_{{cat}}$ values were assigned to the same enzyme for different reactions. All the above works were implemented using both the COBRA [22] and the GECKO toolbox [14], and the model was simulated on MATLAB R2019b by using Gurobi as the optimization solver.

Here, we do not apply additional constraints on those enzymes that no abundance data was available. Because the upper bound constraint of total enzyme concentration measurements as the sum of this portion of the reaction flux is too broad, and we hope to refine the model through more specific constraints of protein on reaction. Combined with the development of absolute quantitative proteomics of *A. niger*, we can easily introduce new protein concentration data into eciJB1325. In addition, the experimental value of total enzyme concentration could be integrated with the model using the function addCouplingConstraint [22], which could couple all the enzymes in the constraint model.

The protein abundance data of *A. niger* can be obtained from the database PAXdb [23]. We retrieved the abundance of 1255 proteins in eciJB1325 with 270 proteins having not found any abundance data. To avoid over-constraint to the model, we selected the constraints for protein abundance as follows. We first matched the maximum of protein abundance value that reported for *A. niger*, and for proteins with no abundance value for *A. niger*, we searched for the homologous proteins’ abundance of organisms in the order of the same genus, the same family, the same order, the same class, and selected the maximum value among them as the upper bound of the reaction flux. According to the rule, 1255 enzymes have been matched and assigned with abundance values as model constraints, and the abundance of core proteome in eukaryotes has been proved to be conservative [24]. In this work, we sought out constrained upper limits of abundance for a total of 985 enzymes respectively, while for the 270 enzymes without abundance matching, we removed their constraints on the model.

### Acquisition and correction of enzyme kinetic parameters

The $k_{{cat}}$ values of all 1255 enzymes in eciJB1325, were primarily derived from the database BRENDA [25]. However, some proteins may have more $k_{{cat}}$ values under different conditions or by different researchers, and some may have no $k_{{cat}}$ published in BRENDA. To tackle this issue, we applied the following strategy to determine the $k_{{cat}}$ value for each of the 1255 enzymes of eciJB1325: for proteins with any $k_{{cat}}$ value founded in BRENDA, the maximum value is used; or if there is specific activity (SA) of the enzyme reported, the $k_{{cat,max}}$ is determined using Eq. (2) The specific activity and the relative molecular weights of the enzymes were from the database UniProt [26].

2 $$ k_{{cat}} \left[ {h^{{ - 1}} \left] { = SA} \right[\frac{{\mu mol}}{{mg*min}}\left] { \times MW} \right[\frac{g}{{mol}}\left] { \times 60} \right[\frac{{min}}{h}} \right] \times 10^{3} \left[ {\frac{{mg}}{g}} \right] \times 10^{{ - 6}} \left[ {\frac{{mol}}{{\mu mol}}} \right] $$

Since the $k_{{cat}}$ values that were automatically matched from the database were too small, the $k_{{cat}}$ values needed to be corrected after incorporating the enzyme constraints as described above. For the metabolic reactions catalyzed by enzymes under the given condition (C), there is a proportional relationship between reaction flux and enzyme concentration [27] (Eq. 3)

3 $$ k_{{app,j}} \left( C \right) = \frac{{v_{j} \left( C \right)}}{{E_{j} \left( C \right)}} = k_{{cat,j}}  \times \eta \left( C \right) $$

The actual catalytic rate of enzyme,$~k_{{app}}$, varies with condition C.$~k_{{cat}}$ is the maximum catalytic rate of enzyme, and η(C) is the condition-dependent efficiency parameter. For each metabolic reaction within the cell, some conditions allow the enzyme to reach its maximum catalytic rate. According to this idea, we simulated the reaction flux $v_{j}$ under multiple conditions and obtained the corresponding $k_{{app}}$, so that $k_{{cat}} ~(k_{{cat}}$ = $k_{{app,max}}$) was obtained, which can be used to correct the enzyme kinetic parameters of the model.

### Model network analysis

We used Cytoscape [28] to illustrate the metabolite interconnection network. Metabolites are shown as nodes in the network. If two metabolites exist in the same reaction, an edge is formed between the two nodes. Enzymes and pseudo-metabolites in eciJB1325 were taken as reactants in metabolic reaction equations, so they also served as nodes as part of the metabolite network and were distinguished by different colors. The metabolites present in different compartments were also assigned by different colors, thus forming an undirected network diagram containing localization information of enzymes, and metabolites. The specific meanings of network parameters are listed as follows (Table 1).

**Table 1:** The relevant parameters of the network and its specific meaning

Parameters	Description
Number of nodes	Number of metabolites in metabolic network, ***n***
Number of edges	Two nodes participating in the same reaction are connected by edges, $\user2{e}_{\user2{n}}$ is the number of edges connected to node ***n***. The number of edges of the network is the sum of $\user2{e}_{\user2{n}} .$
Average number of neighbors	Number of neighbors $\user2{k}_{\user2{n}}$ is the number of nodes that react with metabolite n
Network diameter	The largest distance between two nodes, ***d***
Network radius	The minimum among the non-zero eccentricities of the nodes in the network, ***r***
Characteristic path length	The expected distance between two connected nodes, ***l***
Clustering coefficient	The clustering coefficient $\user2{C}_{\user2{n}}$ of a node ***n*** is defined as $\user2{C}_{\user2{n}} = 2\user2{e}_{\user2{n}} /\left( {\user2{k}_{\user2{n}} \left( {\user2{k}_{\user2{n}} - 1} \right)} \right).$ The network clustering coefficient is the average of the clustering coefficients for all nodes in the network
Network density	The density of interconnected edges between nodes in the network,$\user2{mean}\left( {\user2{k}_{\user2{n}} } \right)/\left( {\user2{n} - 1} \right)$
Network heterogeneity	The tendency of a network to contain hub nodes,$\sqrt {\user2{variance}\left( {\user2{k}_{\user2{n}} } \right)} /\user2{mean}\left( {\user2{k}_{\user2{n}} } \right)$
Network centralization	The concentration of other nodes connected to a node similar to a stellar,$\max \left( {\user2{k}_{\user2{n}} } \right)/\user2{n}$

### Simulation details

#### Flux balance analysis (FBA)

The mathematical analysis of GSMM is usually based on flux balance analysis (FBA), i.e., the mathematical representation of metabolism is based on steady-state mass balance equations (*Sv* = *0*) and a linear programming (LP) problem is solved [29]. eciJB1325 still follows this rule. For the fermentation culture of *A. niger*, the corresponding experimental data obtained from the literature [30] was used.

i. To examine the biomass specific growth rate under different growth conditions, we maximized the biomass growth rate as the objective, and the constraints were the uptake rate of carbon source and oxygen, the secretion rate of products and byproducts, and the production rate of carbon dioxide.
ii. To examine the secretion rate of the product in the chemostat culture, the biomass specific growth rate was fixed to the dilution rate under the chemostat condition, and maximizing the citric acid production rate was taken as the objective. Other measured reaction rates were added as extra constraints to the model.
iii. Similar method was used to examine substrate uptake rate in chemostat culture.
iv. The above three FBA calculations were based on eciJB1325 and compared with the results of iJB1325.

#### Robustness analysis

Different substrate uptake and oxygen supply rates affect the phenotype of cell metabolism, resulting in different biomass specific growth rates. We studied the robustness of the model predicted biomass specific growth rate by varying the glucose and oxygen uptake rates. The implementation of the robustness analysis was as follows: the exchange reaction fluxes of oxygen and glucose were both varying in the range of 0–5 mmol/gDW/h with the objective was set as maximizing the rate of biomass production, and then FBA calculations were performed with the results analyzed.

#### Flux variability analysis (FVA)

The flux distribution calculated by FBA is not unique. Since the degree of freedom of the linear metabolic reaction model is much larger than the given constraints. Even though linear programming can give a single final optimized fluxes results, other fluxes distribution may also give the same maximized biomass specific growth rate [31]. By the FVA method [29], the maximum and minimum values for all reaction fluxes in the model can be determined under certain simulated conditions. The objective function was to maximize the biomass equation with a confidence level of 99.9%. Finally, the flux variability (FV) values of reactions were calculated according to Eq. (4).

4 $$ flux~variablity_{j}  = maxflux_{j}  - minflux_{j} $$

In addition, to measure the effect of integration of enzyme constraints on model flux variability, we calculated the variability reduction of each reaction for eciJB1325 relative to iJB1325(Eq. 5) and the total reduction in variability (Eq. 6).

5 $$ reduction_{j}^{{rxns}}  = \left( {1 - \frac{{flux~variablity_{j}^{{eciJB1325}} }}{{flux~variablity_{j}^{{iJB1325}} }}} \right) \times 100\% $$

6 $$ reduction^{{model}}  = average\left( {reduction_{j}^{{rxns}} } \right) $$

#### Gene knockout simulation

First, we calculated the biomass specific growth rate of the wild-type *A. niger* model under the experimental conditions [30]. Second, single-gene knockout was performed for each of the 1325 genes in the *A. niger* model, and the resulted 1325 mutants were simulated. We simulate the biomass specific growth rates (µ) of the 1325 mutants under the same experimental conditions and calculated the biomass specific growth rate ratio (grRatio) of the mutant to the wild type by Eq. (7), which is then used to determine whether the corresponding knockout gene is necessary, here we take grRatio < 1 as the criteria for this.

7 $$ grRatio_{i}  = \frac{{\mu _{i}^{{mutant}} }}{{\mu _{i}^{{wild}} }} $$

#### Differential expression of enzymes under different carbon sources

To examine the ability of the proposed model, we compared the differential expression of enzymes for *A. niger* under three different kinds of carbon sources: glucose, xylose, or maltose. Expression information of proteins for *A. niger* with xylose and maltose respectively were taken from the literature [32, 33]. When xylose was used as the only carbon source, expressions of the enzymes related to xylose metabolic pathway, arabinose metabolic pathway, β-glucose metabolic pathway, aldehyde reductase, and thiamine synthetic pathway were up-regulated; While when maltose was used as the only carbon source, the expression of glucosidase would be increased accordingly. We modeled these variations on eciJB1325.

i. The flux of glucose exchange reaction of eciJB1325 was set to 1.0 mmol/gDW/h, and the limitation of the oxygen exchange rates was eliminated. The optimization object was to maximize biomass growth rate, and FBA calculated the flux of 1255 enzyme-exchange reactions under the above conditions.
ii. The carbon source condition was changed by limiting the flux of the glucose exchange reaction to zero. To ensure the same number of carbon moles under the different substrate conditions, the flux of the exchange reaction was 1.2 mmol/gDW/h when xylose was the carbon source and 0.5 mmol/gDW/h when maltose was the substrate. At the same time, the oxygen source limitation was removed, and the objective function was to maximize the biomass. Then, the fluxes of 1255 enzyme-exchange reactions under the above two conditions were calculated by FBA.
iii. ii under two conditions relative to i, the differential values in the fluxes of the enzyme-exchange reactions were calculated. The fold changes of enzymes when using xylose and maltose relative to that when using glucose in eciJB1325 were obtained respectively.

## Results

### Model basic information

**Fig. 1:**
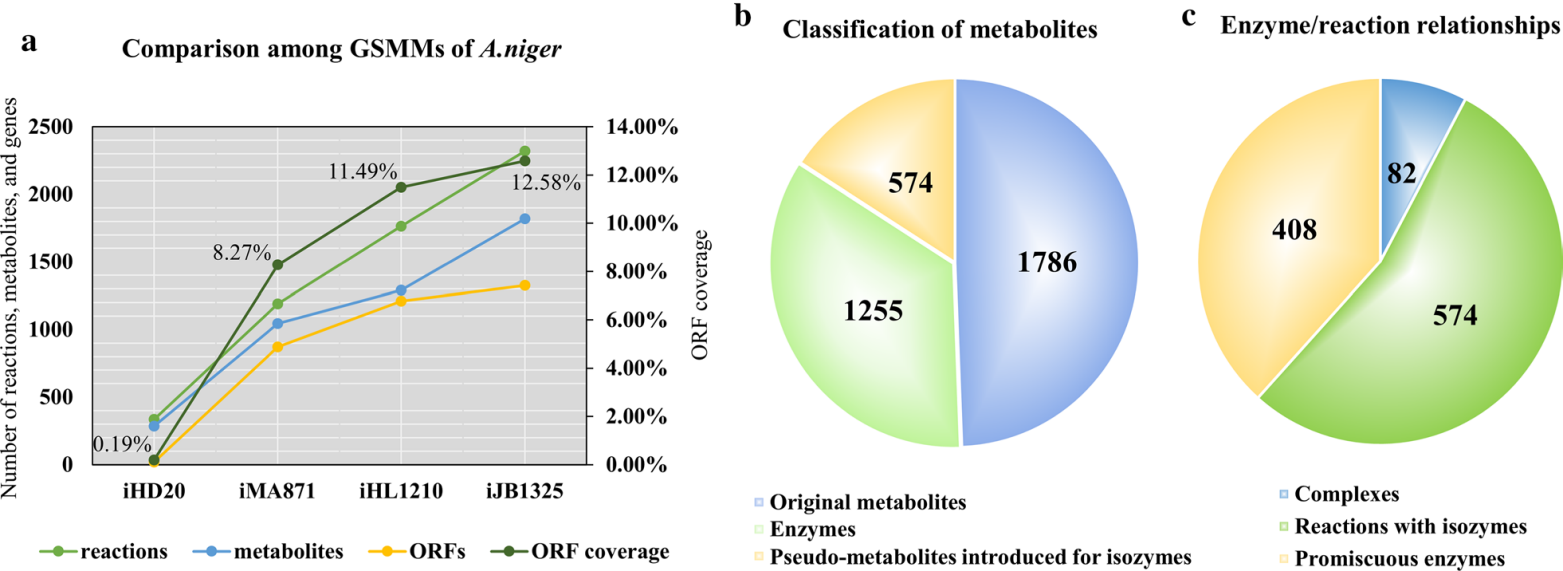
Comparison among GSMMs of *A. niger* (**A**) and classification of metabolites (**B**) and enzymes (**C**) in eciJB1325

Several versions of GSMM model for *A. niger* have been established, which include iHD20 [6], iMA871 [5], iHL1210 [7], and iJB1325 [8]. Properties of these models are compared and shown in Fig. 1A. In this work, we used the up-to-date version iJB1325 as the base for implementing the enzyme constrained GSMM model, called eciJB1325. The new model incorporates 1255 enzyme constraints in total, and it contains 6274 reactions and 3588 metabolites. The newly added metabolites are the 1255 enzymes and 574 pseudo-metabolites introduced for dealing with isozymes (Fig. 1B). Different patterns of enzyme-catalyzed reactions were considered in the model, and different types of enzymes were distinguished (See method for details). Among the 1255 enzymes introduced, 574 were isozymes, 82 were components of enzyme complexes, and 408 were multifunctional enzymes (Fig. 1C, Table 2).

**Table 2:** Comparison between iJB1325 and eciJB1325

	iJB1325	eciJB1325
Number of reactions	2320	6274
Number of metabolites	1818	3588
Number of compartments	7	7
Number of genes	1325	1325
Additional information about eciJB1325		
Metabolic reactions matched with an enzyme(s)		1663
Metabolic reactions not matched with an enzyme		1367
Arm reactions introduced for isozymes		547
Enzyme usages (treated as reactions)		1255

**Fig. 2:**
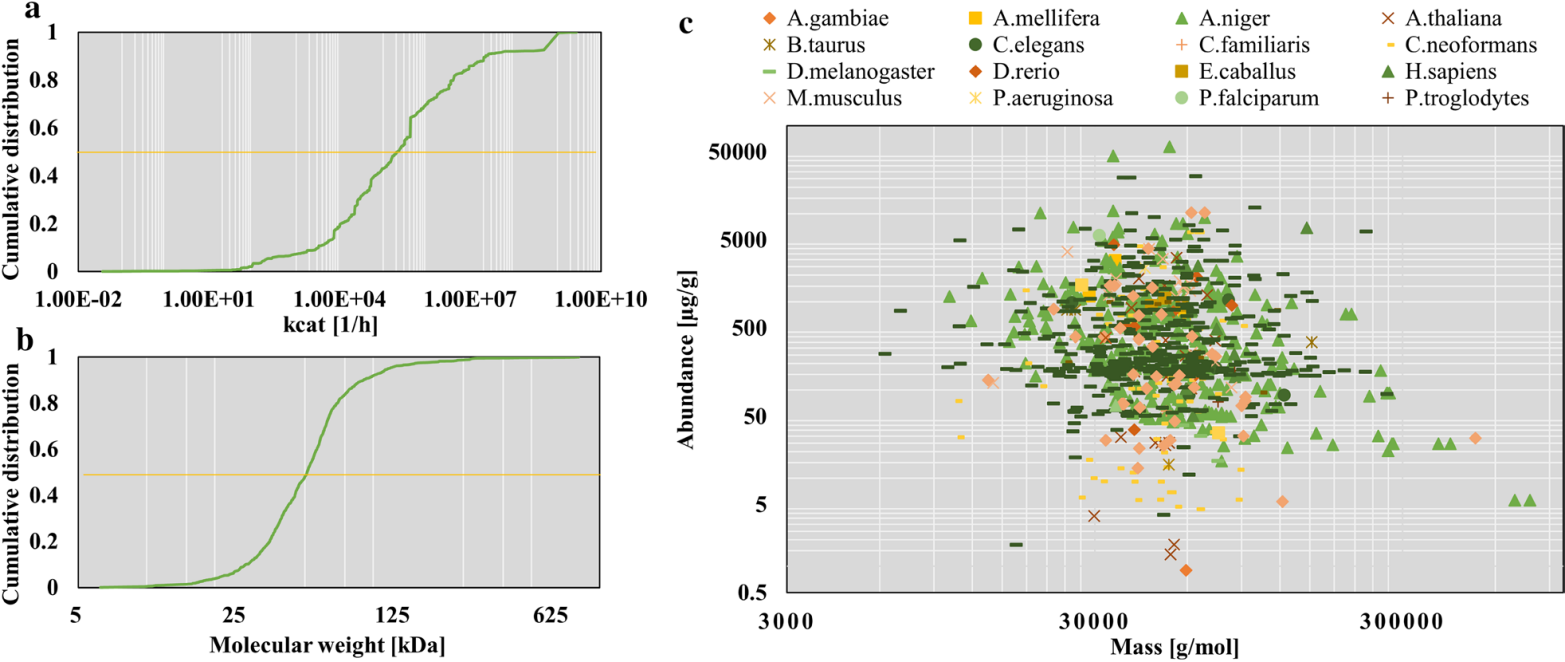
Basic information about enzyme proteins used in the model. **A** Cumulative distribution of *k*_*cat*_ values; **B** cumulative distribution of molecular weights; **C** The abundance information and corresponding molecular weights of 985 proteins collected from 23 eukaryotes

Kinetic parameters ($k_{{cat}}$ value) and molecular weight (MW) of the enzyme were derived from the databases BRENDA [25] and UniProt [26], respectively. Based on the different actions of 1255 enzymes, we matched a total of 2488 $k_{{cat}}$ values under different reaction conditions, and these $k_{{cat}}$ covered 11 orders of magnitude, with a median value of $2.05 \times 10^{{ - 5}}$ h^−1^ (Fig. 2A). The molecular weight values of 1255 enzymes spanned three orders of magnitude with a median of 51.03 kDa (Fig. 2B).

To provide a maximum constraint on the flux of the enzyme participating in the catalysis, we retrieved the enzyme abundance data from the database PAXdb [23]. Since the quantitative protein information of *A. niger* is scarce, we extended our search to homolog proteins in the eukaryote community, and we finally obtained approximate abundance information and corresponding molecular weight values of 985 proteins, derived from 23 eukaryotes (Fig. 2C). Among them, 331 protein abundance information was taken from *A. niger* itself, accounting for 33.60% of the total number, with most of the abundance information was obtained from *Saccharomyces cerevisiae* (45.48%) and part from *Cryptococcus neoformans* (10.66%), which were relatively close to *A. niger*.

### Correction of enzyme kinetic parameters to overcome model over-constraint

**Fig. 3:**
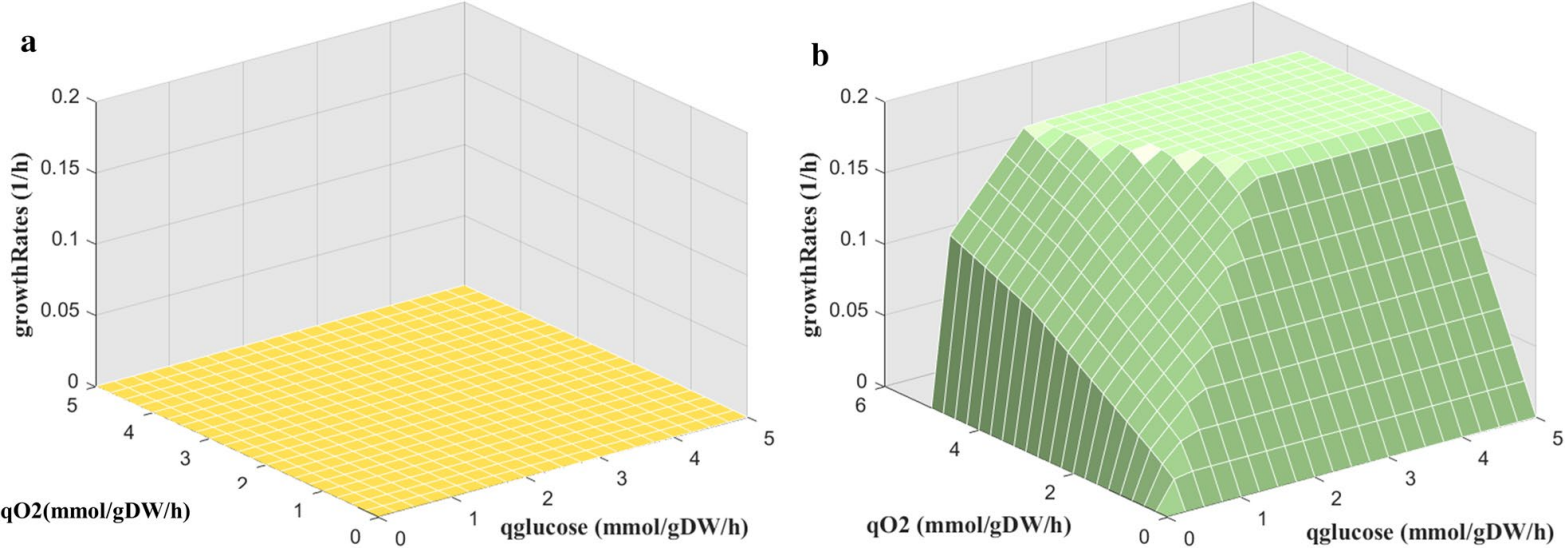
Over-constraint problem with the enzyme constraint model before and after kinetic parameter correction. Direct integration of *k*_*cat*_ values from database sources and iJB1325 results in severe over-constraint of the model. **A** Robustness analysis shows that the model does not show any growth with the increase of nutrient uptake rate; **B** the over-constrain problem was solved by replacing *k*_*cat*_ from database with *k*_*app,max*_ obtained through thoroughly simulation with vast conditions

**Fig. 4:**
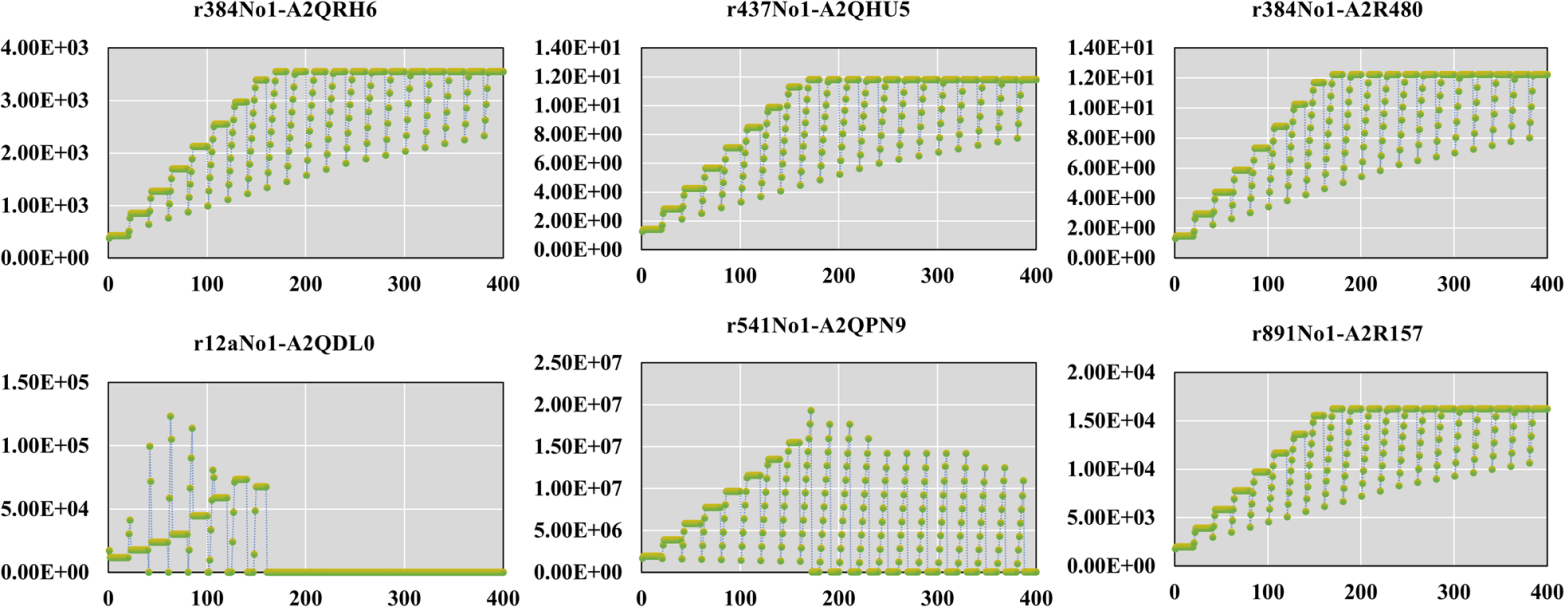
*k*_*app*_ (h^−1^) values varied with conditions. The abscissa represents the simulated 400 conditions, and the ordinate represents the *k*_*app*_ (h^−1^) value for each simulated condition

Through the integration of enzyme kinetic parameters and enzyme abundance information, we built the enzyme-constrained model eciJB1325. However, we found that the model behaved with a very serious over-constraint problem. Some reactions were blocked directly when we checked the results. It is found that these reactions were constrained with too small $k_{{cat}}$ values, which in turn assigned over constraint to the reaction flux resulted in zero growth of the cell (Fig. 3A). To tackle this issue, we derived $k_{{app}}$ under different conditions [27], the maximum $k_{{app,max}}$ among them were used to replace original $k_{{cat}}$ values. The variations of $k_{{app}}$ values with the conditions are shown in Fig. 4. Using this method, $k_{{cat}}$ values was substituted by $k_{{app,max}}$, and the over-constraint problem was overcome (Fig. 3B).

Figure 4 shows how does the $k_{{app,max}}$ is obtained. We simulated 400 conditions through variation of both glucose and oxygen uptake rates, the $k_{{app}}$ values of the enzymes were calculated using Eq. 3. It can be seen that variation of the $k_{{app}}$ values cover as most 1 order of magnitude, but it reached a constant maximum level. Notably, reactions of r384No1, r437No1, and r891No1 exhibited a relatively stable change trend of $k_{{app}}$ values with the changes of simulation conditions, and gradually reached a maximum value. However, the enzymes $k_{{app}}$ values corresponding to reactions r12aNo1 and r891No1 reached the maximum under only a few conditions and then decreased with the changes of conditions. This dissimilarity may be related to the role of isozymes and the path selection of the model.

To calculate $k_{{app}}$, it is hard to obtain corresponding abundance values of the individual enzyme under the 400 conditions. Therefore, the abundance values were fixed rather than condition-specific in the simulation process, and what was actually changing was the magnitude of the fluxes for the different reactions under different conditions. So, the result obtained by this method was only a rough estimate of $k_{{cat}}$ value (Table 3). In addition to the purpose of modifying the model, we also hope to provide some ideas for researchers to dig out the kinetic information of enzymes.

**Table 3:** Rough estimates of $k_{{cat}}$ values and corresponding GPR information

Reaction	EC number	Protein id	kcat from databases (1/h)	Estimated kcat (1/h)
CPAD5P[c] → CO2[c] + H2O[c] + IGP[c]	EC:4.1.1.48	A2QRH6	576.00	3549.02
ADP[c] + RTHIO[c] → DADP[c] + H2O[c] + OTHIO[c]	EC:1.17.4.1	A2QHU5	9.70	11.81
ADP[c] + RTHIO[c] → DADP[c] + H2O[c] + OTHIO[c]	EC:1.17.4.1	A2R480	9.70	12.23
FDP[c] → T3P1[c] + T3P2[c]	EC:4.1.2.13	A2QDL0	21,888.00	123,382.29
AMPm[m] + ATPm[m] → 2 ADPm[m]	EC:2.7.4.10	A2QPN9	179,998.56	4,211,945.08
0.024 C120ACP[c] + 0.013 C140ACP[c] + 0.012 C141ACP[c] + 0.002 C150ACP[c] + 0.154 C160ACP[c] + 0.02 C161ACP[c] + 0.008 C162ACP[c] + 0.002 C170ACP[c] + 0.026 C180ACP[c] + 0.374 C181ACP[c] + 0.327 C182ACP[c] + 0.032 C183ACP[c] + 0.006 C200ACP[c] + MAGLYP[c] → ACP[c] + DAGLYP[c]	EC:2.3.1.20	A2R157	3312.00	16,234.56

The metabolite with [c] presents metabolites in the cytoplasm and [m] in the mitochondria. GSMM also contains compartment information of some metabolites and reactions, which is available in the submitted Additional file 1

### Model network analysis to guide important protein discovery

**Fig. 5:**
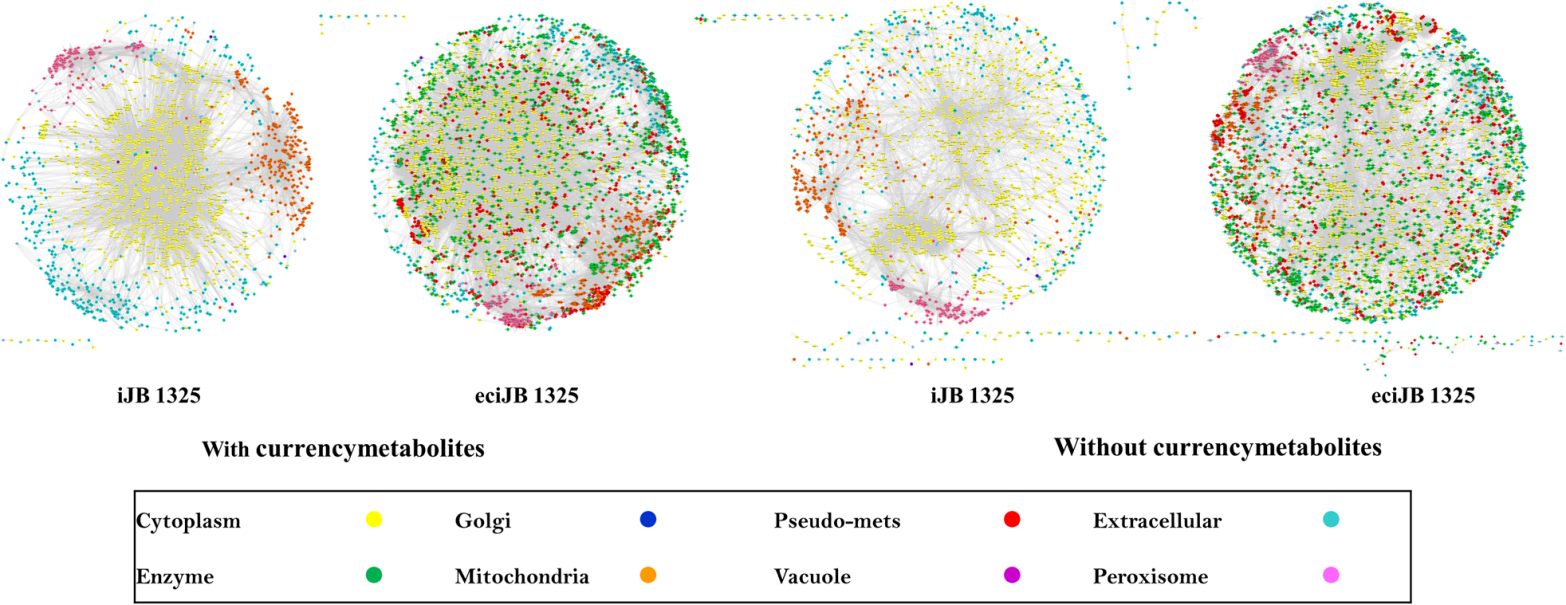
Visualization of metabolic networks for iJB1325 and eciJB1325 (both with and without currency metabolite)

The core of GSMM model is the metabolic network. Metabolite networks are often used to calculate the connectivity of metabolic networks [34–36]. The node of the metabolite network was the metabolite, and two metabolites in the same reaction were connected by an edge. We used Cytoscape [28] to map the undirected networks of metabolites and calculate the related connectivity indexes for both iJB1325 and eciJB1325 (both with and without currency metabolite, Fig. 5), respectively. Although the pseudo-metabolites reduced the clustering coefficient of the network, the addition of other enzymes increased the clustering coefficient of the network (Table 4), so it could be seen that the clustering coefficient of eciJB1325 was increased, and the participation of enzymes also increased the average node degree, network heterogeneity and dispersion of the network, which was caused by the increase of nodes.

**Table 4:** Metabolic network characteristics of iJB1325 and eciJB1325

	With currency metabolites		Without currency metabolites	
	Model	ecModel	Model	ecModel
Number of nodes	1749	3551	1661	3504
Number of edges	20,090	38,258	11,621	24,750
Average number of neighbors	10.046	11.084	6.497	7.849
Network diameter	10	10	15	16
Network radius	5	5	8	8
Characteristic path length	3.27	3.384	4.693	5.115
Clustering coefficient	0.549	0.629	0.354	0.558
Network density	0.006	0.003	0.004	0.002
Network heterogeneity	2.404	2.477	1.753	1.524
Network centralization	0.329	0.259	0.116	0.082

**Fig. 6:**
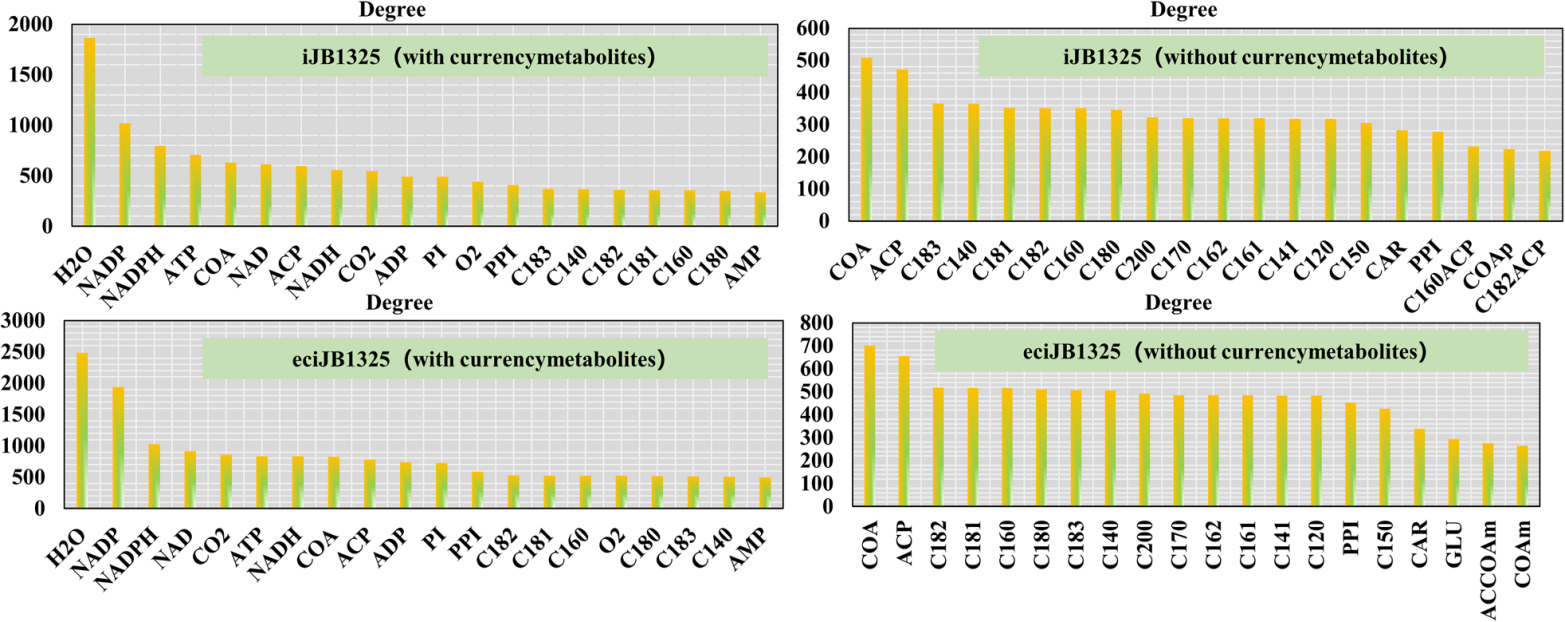
Metabolite connectivity analysis for both iJB1325 and eciJB1325 (with and without currency metabolites, top 20 metabolites at node degree)

For the metabolites with a high node degree in the network, it means they participate in more reactions and often play a more important role in the metabolism. Therefore, we further analyzed metabolite connectivity for both iJB1325 and eciJB1325 by comparing the node degrees of metabolites, and we found that currency metabolites possess the highest node degree value, which means they play vital roles. The node degree of intermediate metabolites in fatty acid synthesis and metabolism is also large in the network because this process always shares the same precursors, e.g., AcCoA. And the connectivity of substances such as glutamic acid/carnitine in the metabolite network was larger which are mainly involved in protein synthesis and fatty acid metabolism (Fig. 6).

**Fig. 7:**
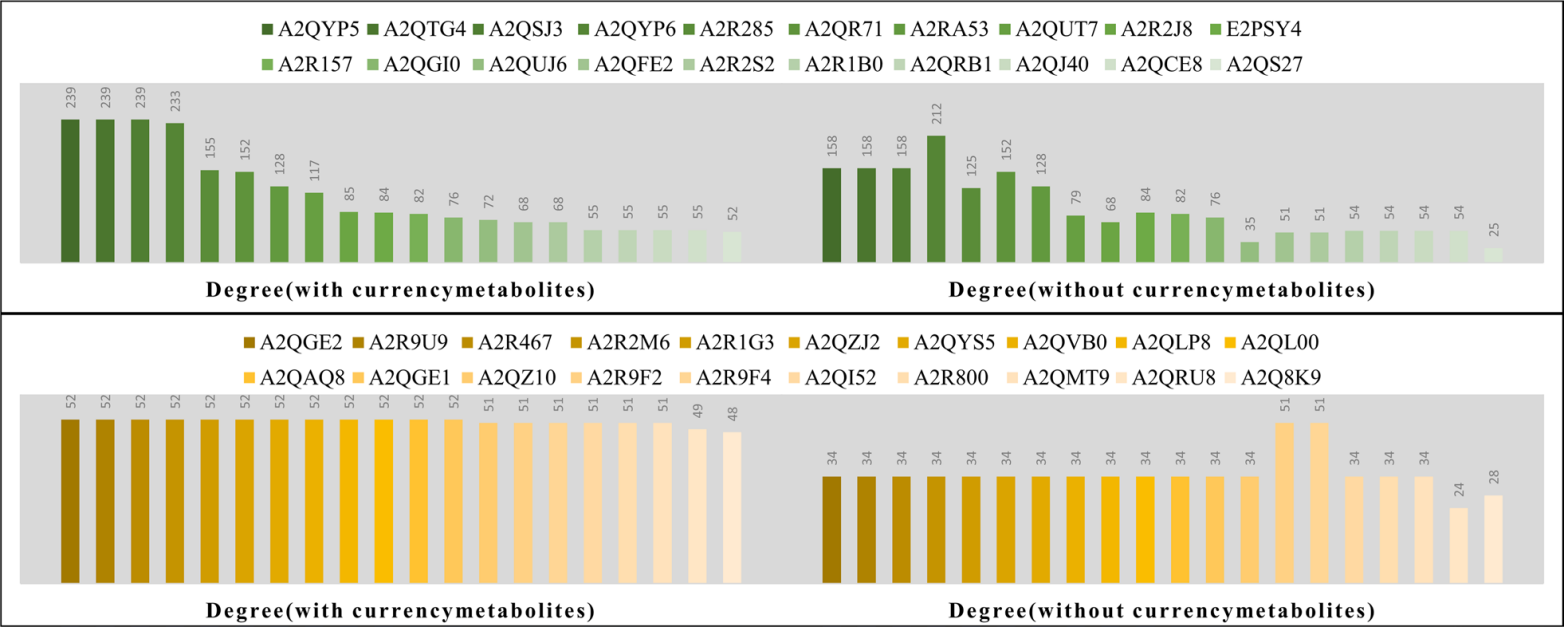
Enzyme connectivity analysis for both iJB1325 and eciJB1325 (with and without currency metabolites, top 20 enzymes at node degree)

As 1255 enzymes in eciJB1325 were considered as reactants, we further analyzed the network connectivity of enzymes, which helped to dissect the importance of different enzymes. We found that enzymes with relatively large connectivity mainly got involved in the fatty acid synthesis and metabolism pathways. These pathways show high connected reactions and multifunctional enzymes (Fig. 7, Table 5).

**Table 5:** Functional matching of 40 proteins with high connectivity

Protein id	Function
A2QYP5	Long-chain fatty acid biosynthetic process
A2QTG4	Long-chain fatty acid biosynthetic process
A2QSJ3	Long-chain fatty acid biosynthetic process/secondary metabolic process
A2QYP6	Fatty acid biosynthetic process
A2R285	Long-chain specific acyl-CoA dehydrogenase
A2QR71	Carnitine *O*-acetyltransferase activity
A2RA53	Cellular lipid metabolic process
A2QUT7	Long-chain fatty acid biosynthetic process
A2R2J8	Fatty acid beta-oxidation
E2PSY4	Carnitine *O*-acetyltransferase activity
A2R157	Glycerol metabolic process/triacylglycerol biosynthesis
A2QGI0	Fatty acid metabolic process
A2QUJ6	CTP biosynthetic process/GTP biosynthetic process/UTP biosynthetic process
A2QFE2	Long-chain fatty acid biosynthetic process/secondary metabolic process
A2R2S2	3-Oxoacyl-[acyl-carrier-protein] synthase activity
A2R1B0	Acetyl-CoA C-acyltransferase activity
A2QRB1	Acetyl-CoA C-acyltransferase activity
A2QJ40	Fatty acid beta-oxidation
A2QCE8	Acetyl-CoA C-acyltransferase activity
A2QS27	Nucleotide catabolic process
A2QGE2	TRANSMEMBRANE transporter
A2R9U9	Short-chain dehydrogenases/reductases (SDR)
A2R467	Short-chain dehydrogenases/reductases (SDR)
A2R2M6	Short-chain dehydrogenases/reductases (SDR)
A2R1G3	Short-chain dehydrogenases/reductases (SDR)
A2QZJ2	Short-chain dehydrogenases/reductases (SDR)
A2QYS5	Short-chain dehydrogenases/reductases (SDR)
A2QVB0	Short-chain dehydrogenases/reductases (SDR)
A2QLP8	Short-chain dehydrogenases/reductases (SDR)
A2QL00	Short-chain dehydrogenases/reductases (SDR)
A2QAQ8	Short-chain dehydrogenases/reductases (SDR)
A2QGE1	Short-chain dehydrogenases/reductases (SDR)
A2QZ10	Fatty acid metabolic process
A2R9F2	Acyl-CoA dehydrogenase
A2R9F4	Acyl-CoA dehydrogenase
A2QI52	Acyl-CoA dehydrogenase
A2R800	Acyl-CoA dehydrogenase
A2QMT9	Acyl-CoA dehydrogenase
A2QRU8	Aldehyde dehydrogenase (NAD+) activity/glyceraldehyde-3-phosphate dehydrogenase (NAD+) (non-phosphorylating) activity
A2Q8K9	ADP biosynthetic process/AMP metabolic process/GTP metabolic process/ITP metabolic process

### Enzyme-constrained integration improves the biomass growth rate prediction

**Fig. 8:**
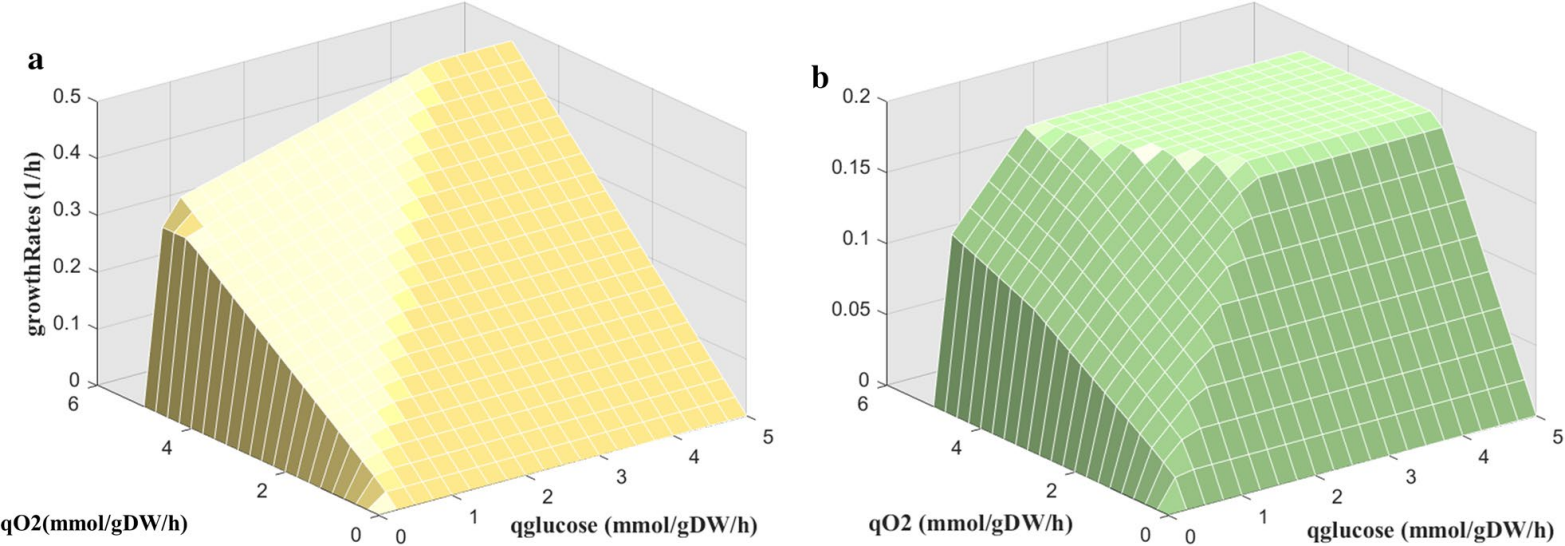
Comparison of robustness results for iJB1325 and eciJB1325. Change in biomass specific growth rate with the increase in glucose and oxygen uptake rate in iJB1325 (**A**) and eciJB1325 (**B**)

One of the main functions of GSMM is to predict microbe behaviors. The main purpose of integrating more information and constraints into the model is to improve the prediction accuracy of the model and enable the model to simulate a more real cell phenotype [37, 38]. Comparing the predicted specific biomass growth rate, product secretion rate, and substrate absorption rate under the condition of chemostat culture with the literature values, we can find that through the integration of enzyme constraints, the prediction accuracy of the model has been significantly improved. The predicted biomass specific growth rate by eciJB1325 was 0.1486 h^−1^, consistent with the literature reported value under the same condition [30], and the prediction error was reduced by 71% compared with that of iJB1325 (with results shown in Table 6). Furthermore, the robustness analysis [29, 39] results of iJB1325 showed that the growth of *A. niger* was not limited in the model with the increase of carbon source and oxygen source, which was not true in reality. However, eciJB1325 simulated a value of 0.1805 h^−1^ for the biomass specific growth rate when glucose was utilized, which was slightly greater than the literature value for the same strain (approximately 0.15 h^−1^) [40]. Therefore, it was acceptable to use this value as the restriction of the critical value of biomass (Fig. 8).

**Table 6:** Comparison of specific biomass growth rate predicted values by iJB1325 and eciJB1325

	Objective	iJB1325	eciJB1325	Literature
Growth rate prediction (h^−1^)	$\max \mu$	0.17	0.1486	0.14 ± 0.01
Substrate absorption rate prediction (mmol/gDW/h)	$\min q\left( {glucose} \right)$	1.5470–1.7084	1.5470–1.8681	1.82–1.84
Product secretion rate prediction (mmol/gDW/h)	${\text{m}}axq\left( {Citrate} \right)$	0.1316–0.3130	0–0.3130	0.02

### Improve model ability for mutation simulation

GSMM has great value on guiding target gene identification for specific property enhancement of strain, such as simulating the change of growth phenotype caused by gene knockout [41, 42]. Individual gene knockout of the 1325 genes for *A. niger* was simulated using iJB1325 and eciJB1325, respectively. The effects of each mutation on biomass specific growth rate of *A. niger* were analyzed. The results showed that eciJB1325 predicted more gene knockouts which can affect cell growth but do not block growth than iJB1325. This indicates the new model improved accuracy for simulating gene mutation than the ordinary model.

**Fig. 9:**
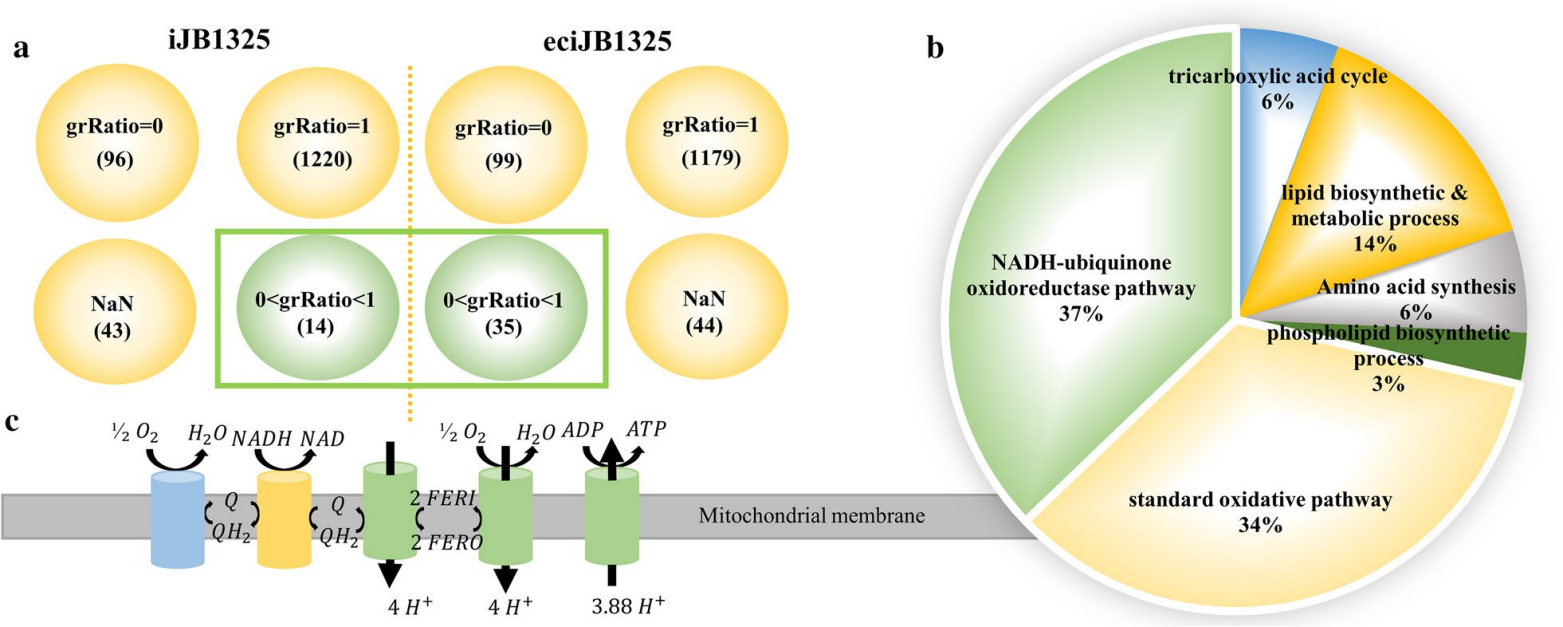
Single knockout of iJB1325 and eciJB1325 leads to phenotypic changes in growth metabolism. **A** The changes of biomass specific growth rate after single knockout of 1325 genes of iJB1325 and eciJB1325 were listed: grRatio = 0, represented that the biomass specific growth rate after gene knockout was zero; grRatio = 1, indicated that gene knockout had no effect on biomass specific growth rate.; 0 < grRatio < 1, represented a decrease in biomass specific growth rate after gene knockout. **B** Functional clustering of enzymes expressed by 35 genes that restrict cell growth but do not cause death. C, Mitochondrial electron transport chain of *A. niger*. The standard respiratory pathway is shown in green, the NADH-ubiquinone oxidoreduction pathway in yellow, and the alternative oxidase pathway in blue

Moreover, eciJB1325 can also explain phenotypic changes at the protein level with growth-related metabolism caused by gene knockout. Further thoroughly investigate the genes identified by eciJB1325, which show growth limiting without blocking effects, get involved mainly in six pathways (shown in Fig. 9B). Among them, the enzymes related to the NADH-ubiquinone oxidoreduction pathway and standard respiratory pathway in the electron transfer chain account for the largest proportion. These enzymes are enzyme complexes and generally regarded as vital for aerobic bacteria, as they supply energy and replenish NADH through electron transfer to oxygen for cell growth. Knockout of these enzymes coded genes will generally cause the death of the cell. However, they showed no death effect by the new model, because *A. niger* has a special alternative oxidase pathway [5] that can supplement part of the electron transport work (Fig. 9C). Deficiency of the normal respiration-related genes causes the decrease of ATP supply, which decreases the cell growth rate, however, the recovery of NADH by the alternative oxidase plays an important role in keeping the cell alive.

Among the identified genes, it also showed that isozymes always stand out. If an isozyme is not expressed due to the knock-out of related genes, other isozymes participated in the catalytic reaction to contribute to cell growth. For example, the effect of knockout of gene coding one enzyme catalyzing tryptophan synthesis (A2R771), can be released by more expression of isozyme gene (An16g02500).

### Enzyme-constrained integration to reduce flux solution space

**Fig. 10:**
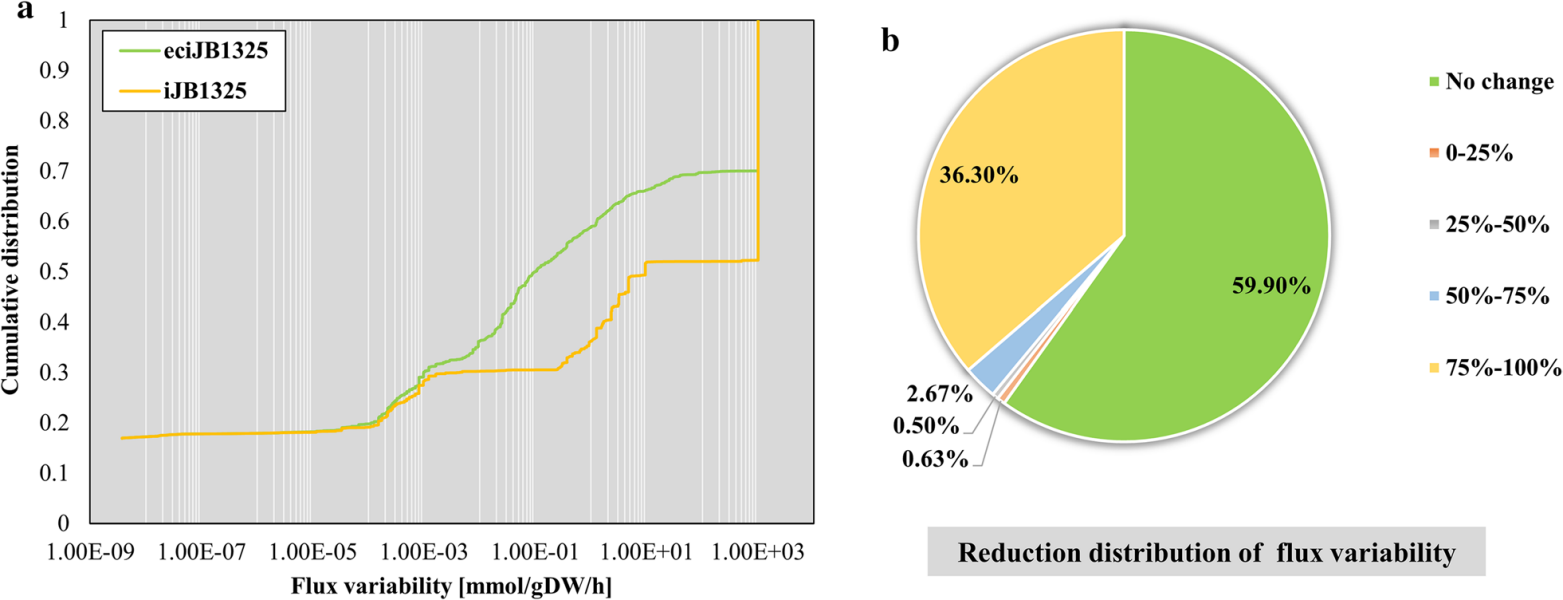
Comparison of flux variability between iJB1325 and eciJB1325. **A** Cumulative distribution of flux variability; **B** Flux variability reduction distribution of eciJB1325 versus iJB1325 reaction

The integration of the enzyme constraints can reduce the flux solution space of the model. We performed a flux variability analysis (FVA) for all reactions of iJB1325 and eciJB1325, respectively, solving for the minimum and maximum fluxes achievable by the reactions and their flux variability (FV, the difference between the maximum flux and the minimum flux). For all the reactions in iJB1325 and eciJB1325, the median of FV in eciJB1325 was much smaller than that in iJB1325, and the total FV of the model was reduced by 37.24% compared with iJB1325, and the distribution of FV also changed significantly (p = 4.4441e−201, Wilcoxon signed-rank test). From the results of reduction in FV of each reaction of eciJB1325 versus iJB1325, the FV of 1215 reactions was reduced (40.10%) and that of 1100 reactions was reduced by more than 75%, accounting for 36.30% of the total number of reactions (Fig. 10B). In summary, the integration of enzyme constraints and GSMM effectively limited the flux solution space of the model and significantly reduced the variability of the model.

### Differential expression of enzymes under different carbon sources

Different carbon sources will affect the composition of secreted proteins of *A. niger* [43]. However, secreted enzymes with different functions will show differential expression for *A. niger* cultured under different carbon sources. Integration of enzyme abundance constraints enables the GSMM ability to simulate this. According to the literature [32, 33], *A. niger* can alter its secreted protein composition when transferred from xylose to maltose condition. For example, when *A. niger* used xylose as the carbon source, the expression levels of xylose metabolic pathway-related enzymes, arabinose-related enzymes, β-glucose metabolism-related enzymes, aldehyde reductase, and thiamine synthesis pathway-related enzymes were up-regulated, and eciJB1325 showed good performance for predicting these up-regulated proteins.

**Fig. 11:**
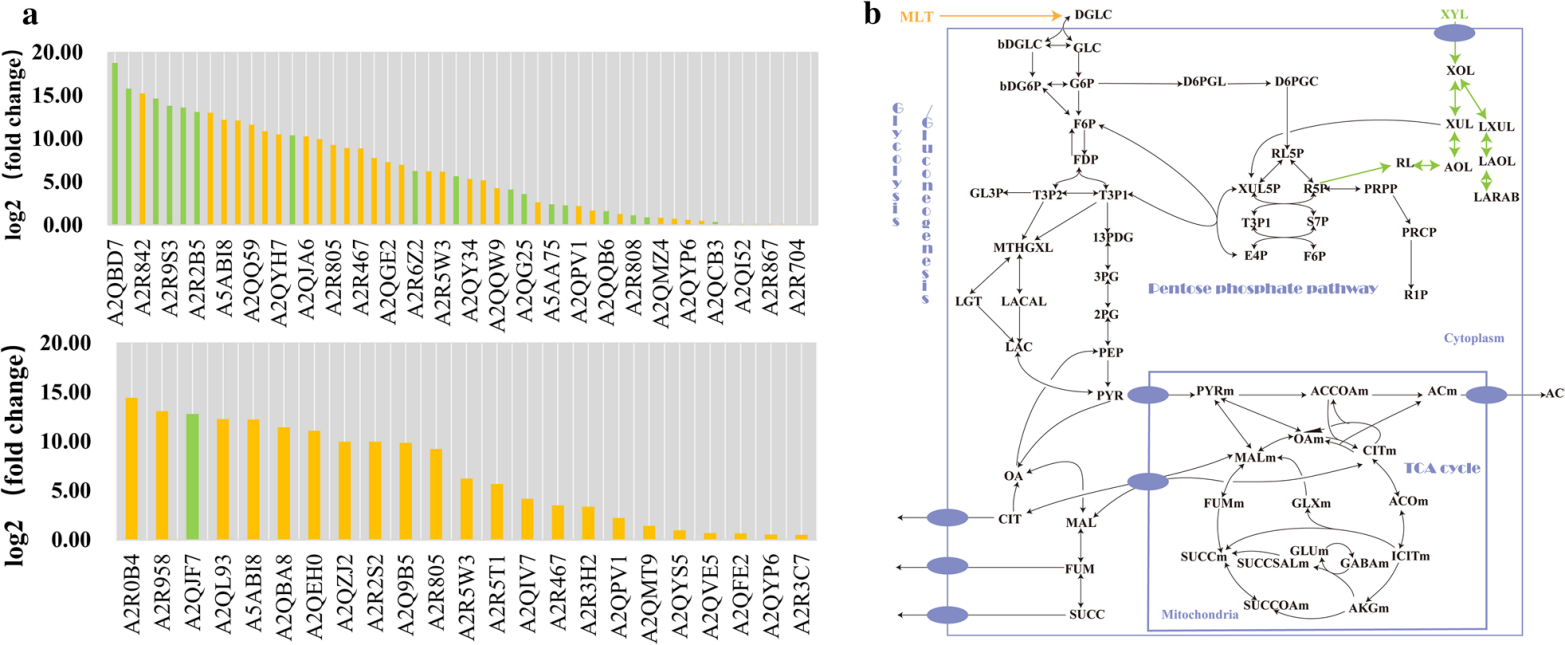
Differential expression of enzymes under different carbon sources. **A** the above picture shows the protein whose expression is significantly up-regulated when using xylose in eciJB1325, among which the green-labeled protein has been reported in the literature; the following picture shows the protein whose expression is significantly up-regulated when using maltose in eciJB1325. The green marked protein is glucosaccharase. **B** the central carbon metabolism pathway of *A. niger* including xylose metabolism and maltose transformation

Among these up-regulated proteins, A2QMS4, A2Q8B5, A2QG25, A2QVE5, and A2QB6 are involved in the xylose metabolism pathway, and A2QB7, A2R6Z2, A2Q8B5, and A2QG25 are enzymes related to arabinose-related enzyme synthesis and metabolism (Fig. 11A). It could be seen from the metabolic pathway diagram of *A. niger* that the xylose metabolic pathway was directly connected with the arabinose metabolic pathway, and they participated in the pentose metabolic pathway of *A. niger*, leading to the further conversion to D-Ribulose involving in pentose phosphate pathway (PPP). Therefore, up-regulation of enzymes related to arabinose synthesis and metabolism could be observed from the model, as well as significant up-regulation of enzymes related to ribose metabolism (A2QBD7, A2R6C9, A2R9S3, A2R2B5, A2QW91, A2QTW0, A2QCB3) (Fig. 11B). Besides, β-glucose metabolism-related enzymes (A2R808), aldehyde reductase (A2QBD7, A2QV34, A2Q8B5, A2QVE5, A2R704), and thiamine synthesis pathway-related enzymes (A2QDB0, A5AA75, A2QRM6) were overexpressed, which was consistent with published data [32, 33].

We also simulated the condition with maltose as a carbon source using eciJB1325. It has been reported that glycosidase is overproduced when *A. niger* utilizes maltose [33]. Model predictions showed a significant up-regulation of Alpha-glucuronidase (aglA/aglU) (Fig. 11A). However, the model failed to simulate the up-regulation of glucose oxidase, superoxide dismutase, and peroxidase reported in [32], which was since the model had too many alternative pathways and lacked kinetic constraints.

## Discussion

Based on the method of GECKO [14], we integrated 1255 enzymes with the genome-scale metabolic model of *A. niger* iJB1325. Unlike small-scale integration that only adds enzyme constraints to several specific reactions [20], eciJB1325 integrates large-scale protein data as constraints, and 1255 enzymes are included covering central carbon metabolism, fatty acid synthesis, amino acid metabolism, and secondary metabolism. In addition, the integration of enzyme constraints does not change the linear structure of the model, but it greatly reduces the available solution space. Comparative analysis of the flux variability between iJB1325 and eciJB1325 reveals that the introduction of enzyme constraints significantly reduced the flux variability.

The integration of enzyme constraints improves the model’s ability to predict metabolic phenotypes. Compared with the original iJB1325, eciJB1325 showed higher accuracy in phenotype prediction, which was shown by closer prediction with cell growth rate, substrate uptake rate, and product secretion rate compared to the experiment results. Especially, robustness analysis showed that the original model predicted an unbounded cell growth rate with excess unlimited nutrients concentration, while the enzyme constraints corrected this. The eciJB1325 gave an upper bound of the cell biomass specific growth rate, 0.181 h^−1^, which is slightly larger than the reported 0.15 h^−1^ [40]. In addition, enzyme constraints enhanced the model's ability to predict potential gene targets for metabolic engineering, e.g., eciJB1325 can identify more growth-related genes through gene knockout simulation, and it can interpret the phenotypic changes caused by gene knockout at the enzyme level. In the new model, alternative isozymes can be activated when one of the isozymes was knock out, e.g., when one enzyme that gets involved in tryptophan synthesis, An16g02500 was knock out, its isozyme, A2R771, takes the responsibility to carry out the corresponding reaction flux to supplement the lacking gene without any harmless to the cell growth. And this cannot be implemented in the original model.

The proposed eciJB1325 model has more practical application values. For example, with quantitative proteomics data, flux upbound of reaction can be accurately simulated and various individual $k_{{cat}}$ value can be conveniently obtained. The enzyme constraints also add a new function to the metabolic network model—predicting the differential expression of enzymes under different growth conditions. According to eciJB1325 simulation results, when the strain uses xylose other than glucose, the expression of xylose metabolic pathway-related enzymes, arabinose-related enzymes, β-glucose metabolism-related enzymes, aldehyde reductase, and thiamine synthetic pathway-related enzymes were up-regulated, which is consistent with the results in the literature [32, 33]. And eciJB1325 also simulated the up-regulation of glucosidase expression when *A. niger* used maltose as substrate [33]. This is valuable as it provides the ability to link environmental conditions and protein expression, as well as the relationship between protein expression and metabolic phenotype.

There is also a limitation for the current eciJB1325 model. Implementation of enzyme abundance constraints depends on accurate kinetic parameters and abundance data of enzymes [14]. However, both of these data *for A. niger* are scarce, here in this work we applied available data of other strains instead for that are missing in *A. niger*. Furthermore, the $k_{{cat}}$ values used in this study are mainly from the BRENDA database [25] and mostly based on in vitro measurements, which may be quite different from in vivo values [27]. From model structure point of view, although the enzyme constraints have greatly improved the predicting ability of GSMM and made the model prediction value closer to experimental measurement, the organism system is too complex and precise to be completely described by only enzyme constraints. So, it is necessary to develop a comprehensive metabolic network model, such as ME model [44], ETFL model [45], etc. Also, the incorporation of molecular structures [46] and unsteady-state dynamics [47, 48] into the reconstructed genome-scale metabolic models is fast-growing, which will open up new prospects for system biology research.

## Conclusions

In this work, we performed large-scale integration of enzyme abundance constraints with the genome-scale metabolic model of *A. niger*, which significantly improved the model's ability to predict metabolic phenotypes and narrowed down the model's flux solution space. The integration of enzyme abundance constraints to GSMM model widened the application of proteomics data in systems biology. With the help of proteomics data, the newly formed model can not only improve the prediction ability but also can be used for detecting target genes for metabolic engineering. The model also showed good ability to be used to estimate the enzyme turn over number ($k_{{cat}}$) with a large-scale manner, and to predict the enzyme expression level under specific growth conditions. This work is an important part of the realization of the comprehensive metabolic network model of *A. niger*, and it also inspires the improvement of genome-scale metabolic models of other strains and promotes the development of GSMM with multi-omics integration.

## Supplementary Information

**Additional file 1.** The enzyme-constrained GSMM of Aspergillus niger (eciJB1325)

## Data Availability

All data generated or analyzed during this study are included in this published article and its supplementary information files.
